# Design, Development, and Deployment of an Electronic Immunization Registry: Experiences From Vietnam, Tanzania, and Zambia

**DOI:** 10.9745/GHSP-D-21-00804

**Published:** 2023-02-28

**Authors:** Emily Carnahan, Linh Nguyen, Sang Dao, Masaina Bwakya, Hassan Mtenga, Hong Duong, Francis Dien Mwansa, Ngwegwe Bulula, Huyen Dang, Maya Rivera, Trung Nguyen, Tuan Ngo, Doan Nguyen, Laurie Werner, Nga Nguyen

**Affiliations:** a PATH, Seattle, WA, USA.; b PATH Vietnam, Hanoi, Vietnam.; c PATH Zambia, Lusaka, Zambia.; d PATH Tanzania, Dar es Salaam, Tanzania.; e National Expanded Program on Immunization, National Institute of Hygiene and Epidemiology, Hanoi, Vietnam.; f National Expanded Programme on Immunisation, Ministry of Health, Lusaka, Zambia.; g Immunization and Vaccine Development Program, Ministry of Health, Community Development, Gender, Elderly and Children, Dar es Salaam, Tanzania.

## Abstract

**Introduction:**

There is growing interest among low- and middle-income countries to introduce electronic immunization registries (EIRs) that capture individual-level vaccine data. We compare the design, development, and deployment of EIRs in Vietnam, Tanzania, and Zambia. Through desk review and the authors’ firsthand implementation experiences, we describe experiences related to timeline, partnerships, financial costs, and technology and infrastructure.

**Implementation Experience:**

The country cases highlight the multi-year timeline required to implement an EIR at scale and the benefit of multiple iterative cycles to pilot and redesign the system before achieving scale. Of the 3 countries, only Vietnam has achieved nationwide scale of the EIR, which took 7 years. In all 3 countries, national government leadership as part of an interdisciplinary team (with experience in leadership, technology, and immunization) was important to ensure country ownership and sustainability. Where international software developers were contracted, partnering with a local software company helped improve responsiveness and sustainability. Across all 3 countries, governments contributed significant in-kind time in addition to investments from donors. Cost savings were observed in Tanzania and Zambia, largely driven by health worker time savings from using the EIR. All 3 case countries underscore the need to understand the local technology and infrastructure context and design the EIR to fit the context. In Vietnam, an initial landscape assessment was conducted to assess technology and infrastructure, whereas in Tanzania and Zambia, user advisory groups provided insights. Existing infrastructure informed EIR design decisions, such as choosing a system with offline functionality in Tanzania and Zambia. All 3 countries have a local partner to provide ongoing technical support.

**Conclusion:**

Comparing implementation factors across these cases highlights practical experience and recommendations that complement existing EIR guidance documents. The findings and recommendations from this study can inform other countries considering or in the process of implementing an EIR.

## Abstract (precis)

We compare experiences implementing electronic immunization registries across countries to highlight findings and recommendations related to time, partnerships, financial costs, and technology and infrastructure.

## Teaser

**Key Findings:**

- Electronic immunization registry (EIR) development benefited from a phased scale-up approach, including multiple system iterations in each country. Although this lengthened the timeline, the process ultimately strengthened the system.
- The role of the software developer was a key difference in partnership approaches. Challenges resulting from working with international developers were addressed by also contracting a local software partner.
- Significant in-kind time contributed by the ministries of health and donor investment enabled these EIR implementations. Unanticipated costs resulted from system technical issues and the need to expand the server as new children are registered.
- The technological infrastructure in each country informed EIR design decisions, with adaptations made for level of computer literacy and Internet connectivity.

**Key Implication:**

- Recommendations to countries implementing an EIR include (1) plan for an iterative development process; (2) establish an interdisciplinary leadership team—including national government staff—with clear roles; (3) ensure funding to sustain and maintain the system; and (4) develop long-term plans for maintenance, updates, and end-user support.

## INTRODUCTION

Immunization is an essential health service provided in all countries. In many low- and middle-income countries (LMICs), immunization information has traditionally been captured through paper-based tools at health facilities.^1^^–^^3^ However, as many LMICs are undergoing digital transformation of their health systems, there is an opportunity to leverage digital health solutions for immunization.^2^^,^^4^ One such tool is an electronic immunization registry (EIR), a confidential, computerized, population-based routine system to capture, store, access, and share individual-level, longitudinal health information on vaccine doses administered.^5^^,^^6^ EIRs have been shown to strengthen immunization practices in LMICs by saving health worker time with automated reporting,^7^^,^^8^ improving data quality and use,^3^^,^^9^^–^^11^ reducing stock-outs,^12^ and improving immunization coverage.^13^^–^^15^ A 2021 report found that EIRs had been piloted or implemented in more than 50 LMICs.^16^

There are several resources that provide practical guidance for practitioners to inform the design, development, and deployment of EIRs.^17^^–^^19^ However, the experience varies by country. Practitioners and researchers have called for more implementation research to understand the “how” and “why” of digital health implementations.^20^^,^^21^ Moreover, the existing guidance on EIRs does not include specific details implementers often want to know related to timelines, costs, human resources, and other operational considerations. There is an opportunity to learn from countries that have experience implementing EIRs to inform other countries considering the same.

Existing guidance on EIRs does not include details related to timelines, costs, human resources, and other operational considerations.

We document the practical experience of scaling an EIR across 3 country contexts: Vietnam, Tanzania, and Zambia. By doing so, we aim to provide practical, operational insights to country decision-makers and partners who are considering or are in the process of implementing an EIR.

## IMPLEMENTATION SETTINGS

In Vietnam, Tanzania, and Zambia, the governments began designing and developing EIRs in the 2010s, making them early adopters of EIRs among LMICs. PATH, an international nongovernmental organization whose mission is to advance health equity,^22^ has been collaborating with and providing technical support to these country governments to introduce and scale their EIRs. Each country immunization context is further described in Table 1. Each country is in the process of scaling an EIR nationwide. Key characteristics of each country’s EIR are captured in Table 2.

**TABLE 1.** Comparison of Country Immunization Contexts in Vietnam, Tanzania, and Zambia

	**Vietnam**	**Tanzania**	**Zambia**
Health system	Vietnam’s health system consists of 5 main levels: national, regional, provincial, district, and commune. The MOH establishes policies, manages national and regional hospitals/institutes, and provides general oversight of the health system.	Tanzania’s health sector operates under the MOHCDGEC, and the President’s Office - Regional Administration and Local Government plays a prominent role in implementations.	Zambia’s MOH provides national policy and technical guidance, which is then interpreted at a provincial level to support hospital and district health management teams.
Immunization program	The NEPI was established in 1985 to provide free immunization services to protect children from the most common infectious diseases. Despite high vaccination coverage, the country faced vaccine data quality challenges, and increasing urbanization and immigration made it challenging to ensure every child received all lifesaving vaccines.	The Immunization and Vaccine Development Program of the MOHCDGEC focuses on administration, M&E, cold chain and logistics, routine immunization, and training.^23^ Despite high vaccination coverage, the country faces challenges related to subnational inequalities in coverage, low uptake of new vaccines, inaccurate target populations, and insufficient data use.^24^	Since the mid-1990s, Zambia has decentralized many health services, including immunization, to its 9 provinces and 72 districts. In recent years, Zambia has improved its immunization service delivery and sustained high coverage rates. However, the country still faces service delivery challenges, including highly mobile populations, vaccine-preventable disease outbreaks, subnational coverage inequalities, and data quality challenges.^25^
Immunization data and reporting	Before nationwide scale-up of Vietnam’s EIR in 2017, most health facilities across the country used paper-based forms to record, manage, and manually plan for immunization delivery, stock management, and reporting. The paper-based forms were often missing data, time consuming and laborious to complete, and a barrier to complete reporting and efficient vaccine stock management.^26^ Now all immunization facilities capture data in the National Immunization Information System. Some facilities have transitioned to paperless reporting (using only the EIR), but most use parallel paper-based and electronic systems.	Before the launch of its EIR in 2016, Tanzania’s immunization information system was paper-based at the facility level and digital from the district level up. At the facility level, health workers recorded monthly reports on paper before submitting them to district managers who entered digital reports into the DHIS2 system. In addition to the DHIS2, the Excel-based District Vaccine Management Tool and the Stock Management Tool supported immunization service delivery.^10^ Where the Tanzania Immunization Registry has been introduced, most facilities are using it in parallel to these paper-based tools. Facilities in 2 regions have transitioned to paperless reporting.	In Zambia, facility health care workers capture immunization data using paper-based forms and submit a monthly report of aggregate data to the district using the HIA2 form.^11^ At the district, data are entered into the DHIS2, the country’s primary health information system.^27^ The facility paper-based forms continue to be the standard and are used in parallel to the Zambia EIR where it has been introduced.
Vaccination coverage of children^a^			
BCG	88%	75%	92%
Third dose of DTP	83%	81%	91%
Birth cohort	1.4 million (2022)^29^	2.3 million (2022)^30^	683,355 (2022)^31^
National digital health market maturity^b^	2	2	2

Abbreviations: BCG, bacille Calmette-Guerin; DHIS2, District Health Information Software 2; DTP, diptheria-tetanus-pertussis; EIR, electronic immunization registry; HIA2, Health Information Aggregation 2; M&E, monitoring and evaluation; MOH, Ministry of Health; MOHCDGEC, Ministry of Health, Community Development, Gender, Elderly and Children; NEPI, National Expanded Program on Immunization.

a World Health Organization/UNICEF estimates from 2021.^28^

b Based on a 5-point scale where 1 is the lowest maturity and 5 is the highest maturity.^32^

**TABLE 2.** Comparison of Electronic Immunization Registries Across Vietnam, Tanzania, and Zambia

	**Vietnam**	**Tanzania**	**Zambia**
System name	National Immunization Information System	Tanzania Immunization Registry	Zambia Electronic Immunization Registry
Technology platform	Bespoke system developed by Viettel	OpenIZ platform (now known as SanteDB, SanteSuite), an open-source immunization management system^33^	OpenSRP, an open-source mobile digital global goods platform^34^
System functions			
Registration of individuals, including demographic information	X	X	X
Registration of vaccination events, including information on administered vaccine	X	X	X
Growth monitoring data			X
Individual monitoring	X	X	X
Aggregate reports	X	X	X
Stock management	X (from national to commune level)	X (facility)	X
Health worker decision support	X	X	X
SMS reminders for clients	X	X	
Automated scheduling and reminders			X
Interoperability/data exchange	Vietnam developed an API based on HL7/FHIR to enable the NIIS to exchange data with other systems since many fee-based immunization facilities and private hospitals have their own systems to track their clients (covering multiple health areas beyond vaccination). The NIIS is also interoperable with a mobile phone application, known as the e-immunization card, that allows parents/clients to access their individual NIIS data.	TImR is integrated with the VIMS, an electronic logistics management information system that tracks vaccine supplies from national level down to district level. An attempt to integrate TImR with the national birth registry compromised the TImR system performance; this challenge is currently being addressed.	ZEIR is in the process of being integrated with DHIS2, the country’s national HIS. ZEIR reports are currently pushed to a cloned version of DHIS2, mirroring the official HIS. ZEIR is integrated with mVacc (mobile vaccination), a community-level RapidPro system that community health workers use to send SMS messages to remind families to bring their children for immunization.
Workflows	Individuals are registered from birth using a unique ID, which is generated by the NIIS. Data are entered by immunization health workers at health facilities, often using computers shared across health areas. Aggregate data reports are generated for users at all levels of the health system to view.	Individuals are registered from birth. A barcode sticker is applied to the child’s health card and scanned as the TImR unique ID. The caregiver’s ID captured in TImR is linked with the national civil registration and vital statistics system. Immunization providers at health facilities enter data using tablets. Data can be captured in offline mode and later synced. Aggregate data reports are generated for users at all levels of the health system to view.	Individuals are registered from birth using a unique ID generated by ZEIR. Immunization providers at health facilities enter data using Android-based tablets. Data can be captured in offline mode and later synced. Aggregate data reports are generated for users at all levels of the health system to view.

Abbreviations: API, application programming interface; DHIS2, District Health Information Software 2; HIS, health information system; HL7/FHIR, Health Level 7/Fast Healthcare Interoperability Resources; ID, identifier; NIIS, National Immunization Information System; SMS, short message service; TImR, Tanzania Immunization Registry; VIMS, Vaccine Information Management System; ZEIR, Zambia Electronic Immunization Registry.

### Vietnam

In 2010, the National Expanded Program on Immunization began working through Project Optimize to improve immunization coverage rates by developing 2 new systems: ImmReg, a digital registry to manage individual vaccination history, and VaxTrak, a digital vaccine stock management tool.^26^ ImmReg and VaxTrak grew from a district-level pilot to a central piece of the country’s health system, as part of the National Immunization Information System (NIIS) launched nationwide in 2017. The NIIS has been shown to increase vaccination coverage and timeliness,^13^ and as of October 2022, the NIIS includes 32.2 million client records and has been implemented in 16,000 facilities (representing nearly all facilities nationwide, including all community health centers but excluding some hospitals and fee-based facilities). In the second half of 2020, the government of Vietnam began transitioning to a paperless immunization system based on the results of a readiness assessment conducted in 2 provinces.^35^

### Tanzania

The Better Immunization Data (BID) Initiative, funded by the Bill & Melinda Gates Foundation, launched in 2013 to improve data quality and data use for immunization.^36^ Through the BID Initiative, representatives from 10 sub-Saharan African countries codesigned common EIR requirements that became the basis for the EIR requirements in Tanzania and Zambia.^37^

Beginning in 2014, Tanzania’s Ministry of Health, Community Development, Gender, Elderly and Children and PATH collaborated to design and implement an EIR. The first iteration, the Tanzania Immunization Information System, was developed in 2014, but due to technical challenges was replaced by a new system—the Tanzania Immunization Registry (TImR)—in 2016.^38^ TImR was rolled out to facilities as part of a larger package of interventions that focused on data quality and data use.^36^ From 2018 to 2019, the Tanga region, (359 facilities) transitioned to completely paperless reporting. Mwanza (328 facilities) and Kilimanjaro (306 facilities) regions transitioned to paperless reporting in April 2021. As of April 2021, TImR includes 1.9 million client records and has been implemented in 3,736 of approximately 6,000 facilities across 15 of 26 regions in mainland Tanzania.

### Zambia

Like Tanzania, Zambia was selected to implement a package of immunization data quality and use interventions through the BID Initiative.^36^ Beginning in 2015, the Zambia Ministry of Health (MOH) partnered with PATH to develop an EIR. The initial system, built on the DHIS2 Tracker platform, did not meet certain requirements for immunization and entailed a lengthy development timeline. In early 2017, Zambia turned to the Open Smart Register Platform (OpenSRP) to develop the Zambia Electronic Immunization Registry (ZEIR).^34^ After piloting ZEIR in early 2017, the registry was rolled out across all health facilities that deliver immunization services in Southern Province.^36^ As of October 2022, ZEIR has been implemented in 29 districts in Southern and Western Provinces, capturing more than 329,000 client records across 596 health facilities. There are plans to continue to scale ZEIR to reach all 2,600 health facilities across 9 provinces nationwide.

## IMPLEMENTATION EXPERIENCE

There are many factors that influence the sustainability and scale of a digital health intervention. The mHealth Assessment and Planning for Scale Toolkit provides a framework for conceptualizing the various “axes” of scale including: groundwork, partnerships, financial health, technology and architecture, operations, and monitoring and evaluation.^39^ Previous studies have assessed the implementation experiences in Tanzania, Vietnam, and Zambia using this toolkit to understand facilitators and barriers to scaling the EIRs.^40^^,^^41^ Previous studies have also summarized the “on the ground” implementation approach used to introduce the EIRs in Tanzania and Zambia,^42^ challenges in sustaining facility use of the EIRs,^43^ and how the data use culture evolved over time.^9^

Although each country’s implementation experience has been well documented, comparing across country implementation experiences can unearth new insights. We compared experiences related to 4 factors: timelines, partnerships, financial costs, and technology and infrastructure. These factors were prioritized based on the authors’ experience implementing EIRs and frequently asked questions from other stakeholders interested in learning from the case countries’ implementation experience. This study draws from relevant documentation, including project reports, communication materials, planning documents, technical guidance documents, and peer-reviewed literature, as well as the authors’ firsthand implementation experience. The authors are MOH staff and PATH staff who supported the EIR implementations.

Comparing across country implementation experiences can unearth new insights related to specific implementation factors.

### Timeline

**FIGURE 1:**
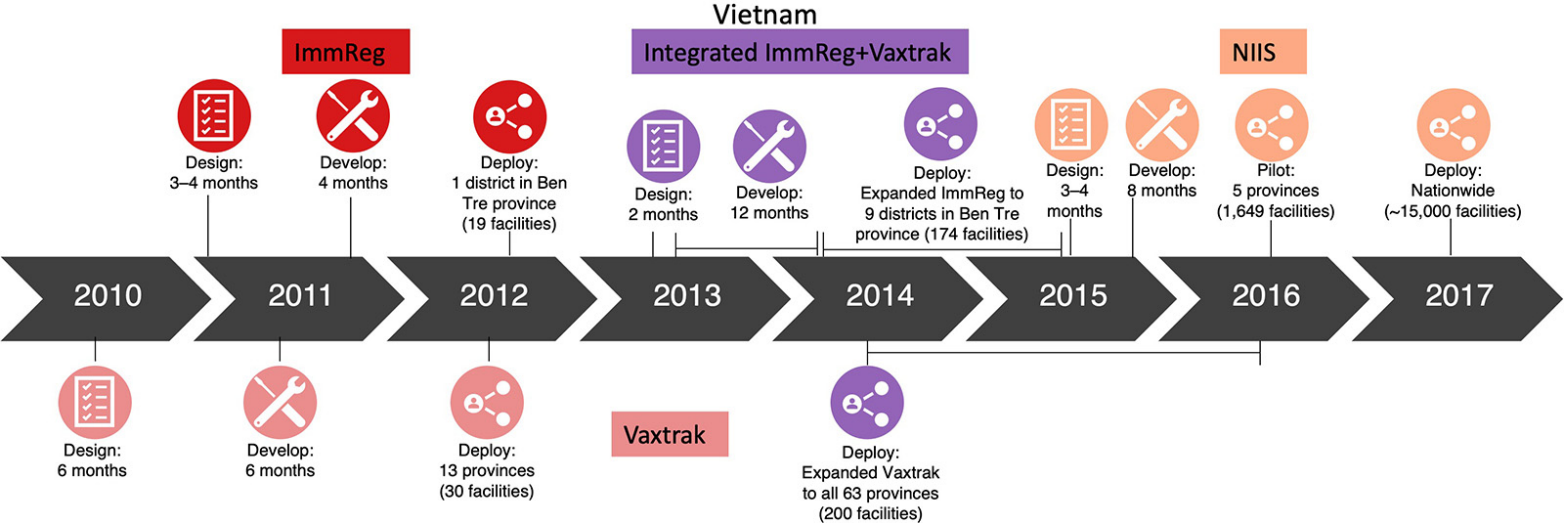
Electronic Immunization Registry Implementation Timelines in Vietnam Abbreviation: NIIS: National Immunization Information System.

**FIGURE 2:**
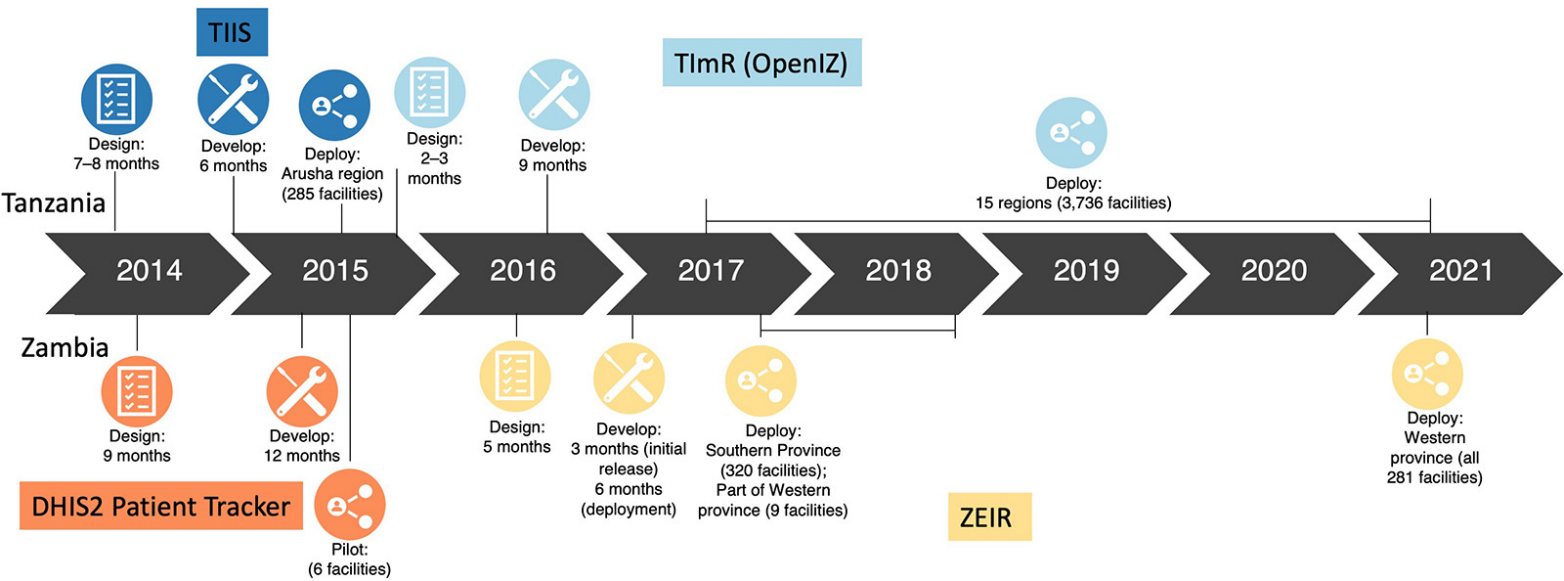
Electronic Immunization Registry Implementation Timelines in Tanzania and Zambia Abbreviations: DHIS2, District Health Information Software 2; TIIS, Tanzania Immunization Information System; TImR, Tanzania Immunization Registry; ZEIR, Zambia Electronic Immunization Registry.

Current EIR guidance documents provide an overview of steps to plan for in the EIR process but do not include details on timelines associated with each step or the overall process as it can vary widely. We considered timing for the key phases of the EIR product development process: design, development, and deployment. Design refers to the initial phase of defining EIR requirements based on user requirements and health system challenges. The development phase refers to building and testing the EIR software based on the predetermined requirements. Finally, the deployment phase refers to the introduction of the EIR system across relevant levels of the health system. Figure 1 and Figure 2 present the detailed timelines for the phases by country.

The cases highlight the long timeline required to implement an EIR at scale. In Vietnam, it took 7 years from the start of Project Optimize^26^ to the nationwide introduction of the NIIS. In Tanzania and Zambia, the EIR development process began with the cross-country collaborative requirements meeting in late 2013, and 9 years later, both countries are still in the process of scaling the EIR nationwide. Across all 3 countries, the timeline included multiple system iterations. Particularly in Vietnam, there was a series of iterative cycles to pilot and redesign the system before achieving scale. This may have lengthened the overall timeline, but each iteration provided an opportunity to redesign and learn from the experience.^41^ Subsequent cycles added more complex requirements, yet the timing for design and development was streamlined because of the ability to build on the existing system. For example, the initial design of ImmReg and Vaxtrak took 4 months and 6 months, respectively, but the integration of ImmReg and VaxTrak into a single web-based application in 2013–2014 took approximately 2 months because the system workflows, interface, functionalities, architecture, and end-user needs were well understood from the previous implementations.

The protracted timeline for EIR design and development in Tanzania and Zambia was in part because the defined requirements were more advanced than the technology available at the time. In Tanzania, development of the Tanzania Immunization Information System was initially planned for 3 months but due to the complexity of the system being developed, it was extended to 6 months. As previously mentioned, technical challenges with the system led to the decision to update the requirements, release a revised request for proposals, and select a new system.^44^

Similarly in Zambia, there were challenges adapting the initial software (DHIS2 Patient Tracker) to meet the requirements, and development stopped after 10 months.^45^ After refining the requirements, Zambia selected the OpenSRP-based ZEIR in early 2017. OpenSRP had already been successfully used for immunization in Pakistan, which contributed to the efficient development in Zambia, requiring 3 months of development time for an initial release and 6 months for a near-complete version for expanded rollout.^46^

The governments of Tanzania and Zambia plan to scale their respective EIRs nationwide; however, there have been delays due to a lack of funding from donors, leadership turnover, and shifts in resources and priorities in response to the COVID-19 pandemic.

### Partnerships

In all 3 case countries, interdisciplinary teams—with experience in leadership, technology, and immunization—were established to oversee the EIR projects, which is consistent with recommendations in existing EIR guidance documents.^17^^–^^19^

In Vietnam, when the NIIS was being redesigned to scale from 1 province to 63 provinces nationwide, the NIIS Technical Working Group (TWG) was formalized. The TWG includes representatives from the following partners.

- The MOH General Department of Preventive Medicine provides management authority, stakeholder coordination, administration support, and oversight of provincial implementation and policy change.
- The National Expanded Program on Immunization provides immunization expertise, acting as the technical lead on immunization workflows, corresponding user requirements, and reporting systems.
- Viettel, one of the largest mobile network operators in Vietnam, provides technology expertise to develop and maintain the system.
- PATH provides technical support and facilitates communication between TWG members, serving as the connection between immunization, general health, and technology stakeholders.

All partners bring complementary expertise and have been crucial to the scale-up of the NIIS from design to deployment. Although the TWG partnership has functioned well, there was initially confusion in roles, deliverables, and financial responsibilities, which created delays in coordination and implementation.^47^ Based on this experience in Vietnam, it would have been helpful to have a memorandum of understanding (MOU) or formal agreement in place to clarify roles, deliverables, financial responsibilities, and outcomes among the TWG partners.^47^

In Vietnam, all partners bring complementary expertise and have been crucial to the scale-up of the NIIS from design to deployment.

In addition, partners at subnational levels in Vietnam were involved in the design phase to inform system requirements, in the development phase to support testing the system and providing inputs on user experience, and in the deployment phase for training and ongoing mentorship. During design and development, representatives of different end-user roles were included, such as managers, Expanded Program on Immunization officers, and storekeepers.

In Tanzania, through the BID Initiative, the Ministry of Health, Community Development, Gender, Elderly and Children partnered with PATH. An MOU was signed in September 2014 to formalize the relationship. PATH provided technical oversight and business analysis support, and the MOH was responsible for testing and ensuring the EIR met the use case requirements. The national Immunization and Vaccine Development Program, the MOH Information Communication Technology Unit, and regional and district leadership (particularly in the pilot site, Arusha Region) were active participants during the design and development phases. During the development phase, Mohawk College (Canada) was selected to develop TImR. Mohawk College partnered with an in-country software firm, Softmed, to provide local support. At the subnational level, a User Advisory Group (UAG) provided end-user perspectives on the system requirements and tested the usability of the system during the design and development phases. UAG members included representatives from regional, district, facility, and community levels who support immunization service delivery, supply chain, data collection, or community engagement.^48^

In Zambia, through the BID Initiative, the Ministry of Community Development, Mother and Child Health partnered with PATH and signed an MOU in 2015 to formalize the relationship. Although the Expanded Program on Immunization unit was under the Ministry of Community Development, Mother and Child Health, the management of the Information Communication Technology Unit and monitoring and evaluation were under the MOH. This structure was challenging to coordinate decision-making between 2 ministries and led to delays in the EIR design, development, and implementation process. A TWG was formed in 2015 that included staff from the Expanded Program on Immunization, Monitoring and Evaluation, and Information Communication Technology units from both ministries which facilitated streamlined decision-making. In 2016, the Mother and Child Health unit shifted to fall under the MOH and a new MOU was signed. In the EIR development phase, Kenya-based software developer Ona was contracted to develop ZEIR and a local software company, BlueCode, was also contracted to support ongoing maintenance of the system. At subnational levels, provincial and district leaders in Southern Province (the area selected to pilot the EIR) worked closely with PATH. Like Tanzania, a UAG was established that included district, facility, and community level end users to provide inputs on the system design and development.^49^

At the national level, all cases highlight the importance of MOH leadership through all EIR phases to ensure country ownership, sustainability, and alignment with national strategies and standards. At subnational levels, all cases highlight the importance of user-centered design to inform the EIR design and development. In Vietnam, this was achieved through an initial landscape assessment to identify critical EIR requirements based on end user needs and by incorporating user feedback and lessons learned in each system iteration. In Tanzania and Zambia, establishing the UAGs was seen as a successful approach to ensure timely testing and input from end users throughout the system design and development.^50^

At subnational levels, all cases highlight the importance of user-centered design to inform the EIR design and development.

A key difference in partnership approaches across the cases is the role of the software developer. In Vietnam, there is a strong public-private partnership where Viettel (mobile network operator) is a formalized member of the TWG and has been actively involved in the EIR design, development, and deployment. This has worked well because Viettel, a state-run organization, has a well-established relationship with the government of Vietnam; the MOH has confidence in Viettel’s data security; and Viettel can support the connectivity, infrastructure, and business needs of the EIR.^47^ Alternatively, in Tanzania and Zambia, the primary technology partners were international organizations that were contracted at the outset of the EIR development phase for a term agreement. In Tanzania, not having the lead developers in-country made communication challenging because of time zone differences and a limited understanding of the local use cases. When there were critical system issues, the fixes were often delayed due to the time zone differences and limited capacity outside of the software teams. Mohawk College partnered with an in-country software firm, Softmed, to improve responsiveness and build capacity for ongoing system maintenance and sustainability. A similar approach was taken in Zambia where Ona partnered with a local software company, BlueCode, which helped address communication, contextualization, and sustainability challenges.

### Financial Costs

EIR guidance documents emphasize the need to develop a funding model that can sustain the project through the entire process, including all organizational costs in the short, medium, and long term.^17^^–^^19^

When the BID Initiative launched in 2013, there was limited evidence on the cost of implementing EIRs—particularly in low-resource settings. Recognizing this evidence gap, the BID team used a total cost of ownership model to estimate the costs to design, develop, deploy, and maintain EIRs in Tanzania and Zambia. This costing work focused on the costs incurred during the duration of the BID Initiative between 2013 and 2018. The results showed that hardware and rollout costs were the largest shares of the overall costs.^51^ However, for each deployment to subsequent regions in Tanzania, costs declined by implementing the learnings gained from previous deployments.

The total annualized cost of developing, deploying, and maintaining the EIRs and their related suite of interventions through the BID Initiative was estimated to be US$3.30–US$3.81 per child for the regions in Tanzania and US$8.46 per child in Zambia.^51^ The Zambia estimate is higher per child in part because of a smaller birth cohort compared to Tanzania. Additionally, a micro-costing study estimated the annual cost savings per facility resulting from the introduction of the BID interventions (including the EIR) as US$10,236 in Tanzania and US$628 in Zambia, largely driven by health worker time savings in service delivery and reporting.^7^ The difference in cost savings between the 2 countries may be because of the shorter evaluation time in Zambia, where health workers may have still been adjusting to the new system post-introduction and had not yet realized its full benefits.

The rollout strategy used in the EIR deployment phase can have large cost implications. Initially in Tanzania, the PATH team led the deployment through a series of visits to health facilities for on-site training. However, this approach was too expensive and technically infeasible to scale. The team shifted to a new approach whereby PATH staff trained district data use mentors who led the facility-level deployment. The new approach improved country ownership and reduced implementation costs by 10%–65% per district.^42^ Vietnam also used a training-of-trainer approach for national, regional, and provincial level staff, as well as training for district staff in 20 provinces. During the deployment phase, user feedback resulted in unanticipated costs to update the NIIS and to provide supportive supervision visits to address technical issues. Overall, the pilot phase in Vietnam was longer than planned, which resulted in higher costs than originally projected.

Across all case countries, the MOH has contributed significant in-kind time, and there was investment from donors, including the Bill & Melinda Gates Foundation, the United Nations Foundation, GlaxoSmithKline, and Gavi, the Vaccine Alliance. In Vietnam, Viettel absorbs many costs associated with the system itself. Since Viettel joined the NIIS TWG, they have provided in-kind services to support the development and deployment of the NIIS. Although Viettel is committed to supporting the NIIS, they have raised the challenge of ongoing investment in infrastructure and human resources.^47^ The Vietnam MOH and Viettel are discussing an appropriate mechanism and service fee that will allow Viettel to operate, maintain, and upgrade the system. In Tanzania and Zambia, the MOH in each country began to take over procurement costs toward the end of the BID Initiative project, including buying tablets, providing project vehicles, and using government staff for trainings.

In all 3 countries, local governments are responsible for budgeting for ongoing operational costs. This includes covering the cost of maintaining or replacing equipment (e.g., computers, tablets, barcode scanners, and printers), conducting refresher trainings, providing supportive supervision, and funding Internet or 3G connectivity. In Vietnam, an ongoing cost that was not factored into the original financial model is the need to update and expand the NIIS server to accommodate the enormity of data as additional children are registered into the system.

### Technology and Infrastructure

EIR guidance documents highlight the importance of considering the infrastructure needs to inform the selection of and support for a new technology.^17^

All 3 case countries underscore the need to understand the local technology and infrastructure context and design the EIR to fit the context. In Vietnam, in the early design phase of ImmReg, a landscape assessment was conducted to assess the information technology knowledge of health workers, lessons learned from previous software implementations, and available equipment and connectivity. The results in 2011 showed that commune health centers did not have computers or Internet connectivity, limited resources were available to purchase computers, and health workers had limited computer skills but were comfortable using mobile phones. Based on this, ImmReg and Vaxtrak were designed as stand-alone mobile applications for commune health workers with a web-based interface for the district level where computers were available. However, despite health workers’ comfort and access to mobile phones, they were not familiar with downloading and installing new software, which created challenges. By 2013–2014, when ImmReg and VaxTrak were combined and upgraded, most commune health centers had computers and an Internet connection, so the combined system was designed as a web-based application.

In Tanzania and Zambia, the UAGs advised on the technological infrastructure during the EIR design phase. In Tanzania, at the outset of the BID Initiative in 2013, eHealth was a national objective and well supported by the President and Minister of Health. The MOH was in the process of establishing an eHealth Steering Committee and eHealth strategy, and there was mainly 2G Internet connectivity in most urban places. However, digital literacy was very low particularly among health care workers, most facilities did not have computers, and some facilities were not connected to the national electricity grid nor Internet services. In Zambia, 144 (48%) of 302 health facilities in the pilot region of Southern Province had no consistent power source.^40^

The existing infrastructure informed decisions during the design phase. In Tanzania, this meant online and offline functionality were required and tablets had to be purchased for most facilities. For facilities without reliable access to electricity, a paper-based version of the EIR was designed that could be scanned at the district level to digitize the data. However, during the pilot phase, it was determined the paper-based forms were not feasible, and all facilities received tablets to use the EIR. Similarly in Zambia, based on the existing infrastructure, the BID Initiative team initially thought that some facilities would need to continue to use improved paper-based registers. However, it was later determined that to ensure cost-effectiveness of the EIR all facilities should use the electronic system. In both countries, solar chargers were procured for facilities with intermittent electricity.

In all countries, the existing infrastructure informed decisions during the design phase.

The deployment phase requires ongoing technical support and system maintenance. In Tanzania and Zambia, there were challenges resulting from limited technology skills and confidence among health workers, difficulty purchasing and distributing sufficient data bundles to support tablet connectivity, and inconsistent data syncing between the tablets and EIR.^40^ There are also ongoing efforts to integrate TImR and ZEIR with other systems in each respective country. In Vietnam, the system has required continuous editing to add new vaccines, new variables, or new data queries based on end-user feedback.

An important aspect of ongoing technical support is direct support to end users. Over time, the approach in Vietnam shifted from in-person to remote technical support, which has saved time, improved responsiveness, and decreased the number of field trips for the technical team. Currently, remote support includes an artificial intelligence chatbot, a telephone hotline, technical support groups on a popular social media application (Zalo), and trained mentors at each level of the health system. In addition to support from the health system, Viettel has an end-user support network and 24/7 hotline that ensure timely technical support for end users. In Tanzania, immunization staff at each level of the health system provide technical support to end users at lower levels, and facility health care workers use district WhatsApp groups to raise issues or troubleshoot. In Zambia, the MOH provides supervision and training and is working with BlueCode to build the MOH capacity for both technical and governance ownership of the project.

## DISCUSSION

The governments of Tanzania, Vietnam, and Zambia were early pioneers among LMICs in Africa and Southeast Asia in designing, developing, and deploying EIRs. After years of implementation efforts, some of the cross-country learnings are just as relevant today, such as the importance of interdisciplinary teams to lead these efforts and the need to design based on the existing technological infrastructure. However, the EIR and digital health context has shifted in important ways.

The protracted timelines for EIR design and development were in part because the systems were newly developed and defined requirements were more advanced than the technology available at the time. Now, the technology has advanced, and there are multiple EIR platforms countries can choose from. For new countries implementing EIRs, the design and development phases should be streamlined compared to our case countries. For instance, OpenSRP software has been used for EIRs in Kenya, Pakistan, and Zambia, with cost and time savings in each subsequent rollout.^46^ And the concise set of requirements used to inform the ZEIR development have been packaged and shared as a proposed minimum viable product for other EIR implementations, regardless of software.^38^ Further, in June 2020, the World Health Organization released a request for proposals to develop a Digital Accelerator Kit that outlines software agnostic requirements for an EIR to accelerate implementation.^52^ These resources will support countries to start from existing software or minimum viable product requirements that can be iteratively adapted or expanded as necessary.

For new countries implementing EIRs, the design and development phases should be streamlined compared to our case countries because the technology has advanced.

Leveraging existing software solutions or requirements has the potential to reduce EIR design and development costs. In the field of digital health more broadly, there has been a call for research on the costs associated with implementation of digital tools, particularly their ongoing operational and maintenance costs.^21^ As more countries introduce EIRs, they should capture their costs to fill this evidence gap and test the hypothesis that there are cost savings from adapting existing systems.

Alongside the availability of new technology, the onset of the COVID-19 pandemic and the subsequent availability of COVID-19 vaccines pushed many countries to consider using digital systems for vaccine management. Many countries adapted systems they were already using. For example, DHIS2 is an open-source health information management system in use in 73 LMICs. Building on their existing systems and capacity in using DHIS2, 43 countries have operationalized DHIS2 as a COVID-19 vaccine management system. Although some of these countries are reporting aggregate data, many of them are using DHIS2 Tracker as an EIR to capture individual-level data on COVID-19 vaccines.^53^

Although not specific to a single implementation factor, the importance of planning for sustainability and scale from the start was an emergent cross-cutting theme. Partnerships that promote government ownership and engage end users can foster sustainability. The importance of planning for ongoing financing, technical support, and system maintenance at scale was also highlighted. Another sustainability consideration that surfaced was planning for the transition from legacy tools, including developing guidance on any criteria that need to be met before making the transition. Other studies have highlighted the burden placed on health workers that are required to use the legacy tools and EIR in parallel.^41^^,^^43^^,^^54^

The Box summarizes the recommendations based on the comparison of experiences in Vietnam, Tanzania, and Zambia. Future research should also compare experiences from other country contexts to add to these recommendations, particularly in Latin America where many countries have implemented EIRs.^55^ And as noted earlier, there are a wide range of factors that influence the sustainability and scale of EIR; in addition to the implementation factors considered in this study, other factors like governance and policy could be considered in future studies.

BOX **Recommendations to Countries Implementing an Electronic Immunization Registry**

- **Plan for an iterative electronic immunization registry (EIR) development process**, starting with the minimum viable product requirements and adapting or expanding the EIR over time. Prepare for multiple pilots or iterations to strengthen the system over the long term.
- **Include end users from the start** to inform the system requirements, support pilot testing, and provide routine feedback to inform incremental system updates.
- **Establish an interdisciplinary team** with experience in leadership, technology, and immunization to oversee the EIR design, development, and deployment. **National government/Ministry of Health staff should lead** to ensure country ownership, sustainability, and alignment with national strategies and standards.
- **Formalize team roles, responsibilities, and commitments** of this interdisciplinary team through a memorandum of understanding or other written agreement.
- **Ensure funding to sustain and maintain the EIR system**, including all recurring costs and software enhancements and maintenance.
- **Capture costs on EIR design, development, and deployment** to fill existing evidence gaps and test the hypothesis that there are economies of scale and cost savings from adapting existing systems.
- **Develop long-term plans for ongoing system maintenance, updates, and end-user support**. This should include identifying local expertise to provide ongoing system maintenance and support beyond the initial deployment.
- **Consider sustainability and scale from the beginning** by establishing government ownership; planning for long-term financing, system maintenance, and user support; and preparing for the transition from the legacy tools to new tools.

### Limitations

This study focused on 3 country cases that were not selected to be representative of countries that have introduced EIRs. Future research could implement a more systematic case selection process with defined criteria to maximize generalizability of the findings from EIR implementations.^56^ Another limitation is this study considered a subset of implementation factors, but taking a wider lens could elicit other findings and recommendations. Finally, the authors acknowledge our participation in the implementation of the EIRs in the case countries. Although this limits the independence of our perspectives, it allows us to draw on our in-depth knowledge of the implementation experience to strengthen the case studies and provide recommendations.

## CONCLUSIONS

As there is growing interest across countries in introducing EIRs to support their immunization programs, they can learn from the experiences of Vietnam, Tanzania, and Zambia. Comparing implementation factors—time, partnerships, financial costs, and technology and infrastructure—across these cases highlights practical experience and recommendations that complement existing EIR guidance documents. As more countries introduce EIRs and leverage existing software solutions, we expect to see further streamlining of timelines and financial costs.

## Notes

**Peer Reviewed**

**First published online:** January 23, 2023.

## References

[1] Namageyo-Funa A Samuel A Bloland P Macneil A. Considerations for the development and implementation of electronic immunization registries in Africa. Pan Afr Med J. 2018;30:81. 10.11604/pamj.2018.30.81.11951. 30344865 PMC6191251

[2] Tozzi AE Gesualdo F D’Ambrosio A Pandolfi E Agricola E Lopalco P. Can digital tools be used for improving immunization programs? Front Public Health. 2016;4:36. 10.3389/fpubh.2016.00036. 27014673 PMC4782280

[3] PATH, Pan American Health Organization (PAHO). *Immunization Data: Evidence for Action. A Realist Review of What Works to Improve Data Use for Immunization, Evidence from Low- and Middle-Income Countries*. PATH/PAHO; 2019. Accessed December 15, 2022. https://www.technet-21.org/en/library/main/5545-a-realist-review-of-what-works-to-improve-data-use-for-immunization

[4] Dumit EM Novillo-Ortiz D Contreras M Velandia M Danovaro-Holliday MC. The use of eHealth with immunizations: an overview of systematic reviews. Vaccine. 2018;36(52):7923–7928. 10.1016/j.vaccine.2018.06.076. 29983255

[5] World Health Organization (WHO). *Classification of Digital Health Interventions v1.0*. WHO; 2018. Accessed December 15, 2022. http://apps.who.int/iris/bitstream/handle/10665/260480/WHO-RHR-18.06-eng.pdf

[6] Freeman VA DeFriese GH. The challenge and potential of childhood immunization registries. Annu Rev Public Health. 2003;24(1):227–246. 10.1146/annurev.publhealth.24.100901.140831. 12668757

[7] Mvundura M Giorgio LD Vodicka E Kindoli R Zulu C. Assessing the incremental costs and savings of introducing electronic immunization registries and stock management systems: evidence from the better immunization data initiative in Tanzania and Zambia. Pan Afr Med J. 2020;35(Suppl 1):11. 10.11604/pamj.supp.2020.35.1.17804. 32373262 PMC7195915

[8] PATH. *PATH Vietnam and ImmReg: Expanding Reach of the Immunization Registry in Vietnam*. PATH; 2016. Accessed December 15, 2022. https://www.path.org/resources/path-vietnam-and-immreg-expanding-reach-of-the-immunization-registry-in-vietnam/

[9] Werner L Seymour D Puta C Gilbert S. Three waves of data use among health workers: the experience of the Better Immunization Data Initiative in Tanzania and Zambia. Glob Health Sci Pract. 2019;7(3):447–456. 10.9745/GHSP-D-19-00024. 31558600 PMC6816809

[10] Kindoli R. *BID Initiative Final Evaluation Report: Immunization Data Quality and Use in Tanzania*. PATH; 2018. Accessed December 15, 2022. http://bidinitiative.org/wp-content/uploads/BID-Initiative_ME-Endline-Report_Tanzania_FINAL_20August2018.pdf

[11] Zulu C. *BID Initiative Midline Report, Zambia.* PATH; 2018.

[12] Gilbert SS Bulula N Yohana E. The impact of an integrated electronic immunization registry and logistics management information system (EIR-eLMIS) on vaccine availability in three regions in Tanzania: a pre-post and time-series analysis. Vaccine. 2020;38(3):562–569. 10.1016/j.vaccine.2019.10.059. 31706808 PMC6983926

[13] Nguyen NT Vu HM Dao SD Tran HT Nguyen TXC. Digital immunization registry: evidence for the impact of mHealth on enhancing the immunization system and improving immunization coverage for children under one year old in Vietnam. mHealth. 2017;3:26. 10.21037/mhealth.2017.06.03. 28828373 PMC5547172

[14] Uddin MJ Shamsuzzaman M Horng L. Use of mobile phones for improving vaccination coverage among children living in rural hard-to-reach areas and urban streets of Bangladesh. Vaccine. 2016;34(2):276–283. 10.1016/j.vaccine.2015.11.024. 26647290 PMC4807732

[15] Chen L Du X Zhang L. Effectiveness of a smartphone app on improving immunization of children in rural Sichuan Province, China: a cluster randomized controlled trial. BMC Public Health. 2016;16(1):909. 10.1186/s12889-016-3549-0. 27581655 PMC5006404

[16] PATH. *Digital Square Electronic Immunization Registries in Low- and Middle-Income Countries*. PATH; 2021. Accessed November 17, 2022. https://static1.squarespace.com/static/59bc3457ccc5c5890fe7cacd/t/60aee1bfd163646306fb924c/1622073794356/Digital+Square+EIR+Landscape_Final.pdf

[17] Pan American Health Organization (PAHO). *Electronic Immunization Registry: Practical Considerations for Planning, Development, Implementation, and Evaluation*. PAHO; 2017. Accessed December 15, 2022. http://iris.paho.org/xmlui/bitstream/handle/123456789/34865/9789275119532_eng.pdf

[18] European Centre for Disease Prevention and Control (ECDC). *Designing and Implementing an Immunisation Information System: A Handbook for Those Involved in the Design, Implementation or Management of Immunisation Information Systems*. ECDC; 2018. Accessed December 15, 2022. https://repository.immregistries.org/resource/designing-and-implementing-an-immunisation-information-system-a-handbook-for-those-involved-in-the-d/from/major-iis-topics/CDC/

[19] World Health Organization (WHO), PATH. *Planning an Information Systems Project: A Toolkit for Public Health Managers*. PATH; 2013. Accessed December 15, 2022. https://www.path.org/resources/planning-an-information-systems-project-a-toolkit-for-public-health-managers/

[20] World Health Organization (WHO). *WHO Guideline: Recommendations on Digital Interventions for Health System Strengthening*. WHO; 2019. Accessed April 11, 2020. https://www.who.int/publications/i/item/9789241550505 31162915

[21] Labrique A Vasudevan L Weiss W Wilson K. Establishing standards to evaluate the impact of integrating digital health into health systems. Glob Health Sci Pract. 2018;6(Suppl 1):S5–S17. 10.9745/GHSP-D-18-00230. 30305335 PMC6203412

[22] PATH. Accessed December 15, 2022. https://www.path.org/

[23] The United Republic of Tanzania. Ministry of Health and Social Welfare (MOHSW) - Tanzania Mainland. *Expanded Programme on Immunization 2010 - 2015 Comprehensive Multi Year Plan*. MOHSW; 2011. Accessed December 15, 2022. https://bidinitiative.org/wp-content/uploads/1405554135TanzaniaComprehensivemultiyearplanfor20102015Year2011.pdf

[24] Gavi, the Vaccine Alliance. *Tanzania Joint Appraisal Update Report 2019*. Gavi, the Vaccine Alliance; 2019. Accessed December 15, 2022. https://www.gavi.org/sites/default/files/document/2020/Tanzania%20Joint%20Appraisal%202019.pdf

[25] Gavi, the Vaccine Alliance. *Zambia Joint Appraisal Report 2018*. Gavi, the Vaccine Alliance; 2018. Accessed December 15, 2022. https://www.gavi.org/sites/default/files/document/joint-appraisal-zambia-2018pdf.pdf

[26] PATH, National Expanded Programme on Immunization. *Summary of Optimize Activities Conducted With Vietnam’s National Expanded Programme on Immunization*. PATH; 2012. Accessed December 15, 2022. https://www.path.org/resources/summary-of-optimize-activities-conducted-with-vietnams-national-expanded-programme-on-immunization/

[27] Zambia HMIS. Digital Health Atlas. Updated March 31, 2020. Accessed December 15, 2022. https://digitalhealthatlas.org/en/-/projects/1126/published

[28] Immunization coverage estimates dashboard. WHO/UNICEF Estimates of National Immunization Coverage (WUENIC) Analytics. July 2022. Accessed December 15, 2022. https://data.unicef.org/resources/immunization-coverage-estimates-data-visualization/

[29] Vietnam. Gavi, the Vaccine Alliance. 2022. Accessed December 15, 2022. https://www.gavi.org/programmes-impact/country-hub/west-pacific/vietnam

[30] Tanzania, UR. Gavi, the Vaccine Alliance. 2022. Accessed December 15, 2022. https://www.gavi.org/programmes-impact/country-hub/africa/tanzania-ur

[31] Zambia. Gavi, the Vaccine Alliance. 2022. Accessed December 15, 2022. https://www.gavi.org/programmes-impact/country-hub/africa/zambia

[32] Herrick T Gannon S Gilbert S. How digital health maturity can inform global goods design. Digital Square blog. December 9, 2019. Accessed December 15, 2022. https://digitalsquare.org/blog/2019/12/6/how-digital-health-maturity-can-inform-global-goods-design

[33] SanteSuite. Accessed December 15, 2022. http://openiz.org

[34] OpenSRP. Accessed December 15, 2022. http://smartregister.org

[35] Duong H Dao S Dang H. The transition to an entirely digital immunization registry in Ha Noi Province and Son La Province, Vietnam: readiness assessment study. JMIR Form Res. 2021;5(10):e28096. 10.2196/28096. 34694232 PMC8576599

[36] Better Immunization Data Initiative. *The BID Initiative Story: Improving Health Services through Innovation in Data Quality and Use.* PATH; 2018. Accessed December 15, 2022. https://bidinitiative.org/wp-content/uploads/BIDStory_PRINT_English_R1_clickable.pdf

[37] Better Immunization Data Initiative. *Product Vision for the Better Immunization Data (BID) Initiative.* PATH; 2014. Accessed December 15, 2022. https://bidinitiative.org/wp-content/uploads/FINAL_BID-Product-Vision-09122014.pdf

[38] Seymour D Werner L Mwansa FD. Electronic immunization registries in Tanzania and Zambia: shaping a minimum viable product for scaled solutions. Front Public Health. 2019;7:218. 10.3389/fpubh.2019.00218. 31440494 PMC6693385

[39] World Health Organization (WHO), United Nations Foundation, Human Reproduction Programme, Johns Hopkins University Global mHealth Initiative. *The MAPS Toolkit: MHealth Assessment and Planning for Scale.* WHO; 2015. Accessed December 15, 2022. https://apps.who.int/iris/handle/10665/185238

[40] Dolan SB Alao ME Mwansa FD. Perceptions of factors influencing the introduction and adoption of electronic immunization registries in Tanzania and Zambia: a mixed methods study. Implement Sci Commun. 2020;1:38. 10.1186/s43058-020-00022-8. 32885195 PMC7427960

[41] Dang H Dao S Carnahan E. Determinants of scale-up from a small pilot to a national electronic immunization registry in Vietnam: qualitative evaluation. J Med Internet Res. 2020;22(9):e19923. 10.2196/19923. 32960184 PMC7539163

[42] Mtenga H Mphuru A Seymour D Werner L. The challenges of implementing a data use culture. MMS Bulletin. 2018;(148). Accessed December 15, 2022. https://www.medicusmundi.ch/de/bulletin/mms-bulletin/digital-health/der-einsatz-digitaler-medien/the-evolution-into-government-led-implementation-of-data-quality-and-use-interventions

[43] Carnahan E Ferriss E Beylerian E. Determinants of facility-level use of electronic immunization registries in Tanzania and Zambia: an observational analysis. Glob Health Sci Pract. 2020;8(3):488–504. 10.9745/GHSP-D-20-00134. 33008860 PMC7541123

[44] Better Immunization Data (BID) Initiative. *Functional Requirements Document for Tanzania Immunization Registry (TImR)*. BID Initiative; 2015. Accessed December 15, 2022. http://bidinitiative.org/wp-content/uploads/TimR-Functional-Requirements_.pdf

[45] Titlestad OH Seymour D. Lessons learned in electronic immunization registry development. Better Immunization Data Initiative blog. November 23, 2016. Accessed December 15, 2022. https://bidinitiative.org/blog/lessons-learned-in-electronic-immunization-registry-development/

[46] Better Immunization Data Initiative, Interactive Research & Development, International Training and Education Center for Health. *The Catalytic Potential of Rapid, Iterative Software Development: Software Lessons From Pakistan, Zambia, and Kenya Lay Groundwork for Successful Electronic Immunization Registries*. PATH; 2020. Accessed December 15, 2022. https://bidinitiative.org/wp-content/uploads/CVIA_BID_Story2019_v1_rev03.pdf

[47] PATH. *Mobile Network Operator Partnerships in Action for Health: A Vietnam Case Study on Mobile Network Operator and Ministry of Health Engagement for Electronic Immunization Registry Application*. PATH; 2019. Accessed June 10, 2020. https://bidinitiative.org/wp-content/uploads/PartneringwMNOsIDEAL-002.pdf

[48] Mwanyika H. Capturing the end user perspective: BID’s User Advisory Group. Better Immunization Data Initiative blog. August 13, 2014. Accessed December 15, 2022. https://bidinitiative.org/blog/capturing-the-end-user-perspective-bids-user-advisory-group

[49] Njobvu FS. User Advisory Group established in Zambia. Better Immunization Data Initiative blog. December 24, 2014. Accessed December 15, 2022. https://bidinitiative.org/blog/user-advisory-group-established-in-zambia/

[50] Msangi M. Coordinated, user-centered approach. Better Immunization Data Initiative blog. September 23, 2015. Accessed December 15, 2022. https://bidinitiative.org/blog/a-coordinated-user-centered-approach/

[51] Mvundura M Di Giorgio L Lymo D Mwansa FD Ngwegwe B Werner L. The costs of developing, deploying and maintaining electronic immunisation registries in Tanzania and Zambia. BMJ Glob Health. 2019;4(6):e001904. 10.1136/bmjgh-2019-001904. 31803511 PMC6882552

[52] World Health Organization (WHO). *Request for Proposals: Immunization Registry Digital Accelerator Kit and Computable Content*. WHO; 2020.

[53] DHIS2 In Action. DHIS2. Accessed December 15, 2022. https://dhis2.org/in-action/

[54] Better Immunization Data Initiative, PATH. *BID Initiative Lessons Learned Encyclopedia*. PATH; 2018. Accessed December 15, 2022. https://bidinitiative.org/wp-content/uploads/FINAL_LessonsLearned_Encyclopedia_6July2018-Logo-1.pdf

[55] Danovaro-Holliday MC Ortiz C Cochi S Ruiz-Matus C. Electronic immunization registries in Latin America: progress and lessons learned. Rev Panam Salud Publica. 2014;35(5–6):453–457. 25211576

[56] Mookherji S LaFond A. Strategies to maximize generalization from multiple case studies: lessons from the Africa Routine Immunization System Essentials (ARISE) project. Evaluation. 2013;19(3):284–303. 10.1177/1356389013495212

